# Struggling for epistemic and emotional justice—a collaborative autoethnography of personal assistance

**DOI:** 10.3389/fsoc.2025.1425224

**Published:** 2025-07-11

**Authors:** Lill Hultman, Maya Hultman

**Affiliations:** Department of Social Work, Marie Cederschiöld University, Stockholm, Sweden

**Keywords:** epistemic injustice, emotional injustice, personal assistance, collaborative autoethnography, crip time, linked lives, emotion work, emotional labor

## Abstract

The present article explores the intersection between disability and the emotions evoked by the experience of living with Personal Assistance (PA) in everyday life. The aim is to explore the emotion work around navigating the emotional and epistemic injustice faced by disabled people and their family members. As family members, mother and daughter, we are bound by our mutual experiences of being recipients of disability support. Research tends to focus on the professional gaze. Hence, the emotion management of disabled people living with disability support and their family members needs to be better understood. Life with PA provides a context that illustrates what epistemic and emotional injustice in various forms feels like. Our narratives may help to increase the understanding of the complex interplay between assistance coordinators, external personal assistants, young adults in need of PA, and family members involved in providing PA in everyday life. Focusing on our experiences of having linked lives underlines the entanglement of having different roles vis-a vis each other. Utilizing a collaborative autoethnographic approach we have identified three themes, *The interconnectedness between emotion invalidation and crip time, The expectation of emotion work* and *Managing conflicting needs in the light of emotion work and linked lives*. The findings show a difference concerning the expectation of emotion management, where external PAs perform emotional labor during work hours, while assistance users and family members perform emotion work throughout the day. Professionals often cause epistemic injustice in different situations and increase the need to perform emotion work in implementing PA instead of acknowledging the lived experience of assistance users and family members. When assistance coordinators or external PAs seek to eliminate certain emotions from the experiences of users or their family members, they overlook valuable insights about the situation. Silencing those with lived experiences risks dismissing individuals who possess relevant first-hand knowledge due to their emotional connection to the experienced injustice.

## 1 Introduction

Epistemic injustice (Fricker, 2007) is concerned with forms of unfair treatment that relate to issues of knowledge, understanding, and participation in communicative practices (Kidd and Carel, 2017) in which the voices and experiences of marginalized individuals are not being taken seriously (Cummings et al., 2023). Emotional injustice occurs when the treatment of emotions is unjust, or emotions are used to treat people unjustly (Pismenny et al., 2024). The psycho-emotional aspects (Reeve, 2002; Thomas, 1999), “work” and “performances” of the “disabled” identity are themes explored within disability studies (Goodley, 2010). To some extent, “emotion work” and “emotional labor” have been explored regarding disabled people's experiences (see for example, Liddiard, 2014; Goodley et al., 2018). Emotion management is both an inner process and an outward expression, frequently involved in preserving social bonds and social rules (Williams, 2003). As such, it becomes relevant for disabled people with PA. This article addresses epistemic and emotional injustices experienced by people living in Sweden with PA in everyday life. We want to underline the difference between the expectations of professionals and service users in handling the emotional aspects of PA, since discarding emotions profoundly impacts both emotional and epistemic injustices. In this article, the aim is to explore the emotion work around the navigation of emotional and epistemic injustice faced by disabled people and their family members, evoked by our experiences of living with PA as a mother and daughter. The former being a parent and the latter a young disabled female PA user.

The first part of the article is mainly theoretical, and the second part is empirical, based on autoethnographic narratives related to lived experiences with PA in our everyday life. We draw on notions of epistemic injustice (Fricker, 2007), emotional injustice (Pismenny et al., 2024), emotion management (Hochschild, 2012) and crip time (Kafer, 2013) to make sense of our autobiographical experiences of living with PA in Sweden, where our experiences of emotion management underline the complex interplay between emotional and epistemic injustice.

PA is a consumer-directed support where disabled people are in control of recruiting, training, and managing the people who support them (Porter et al., 2020). PA differs from other forms of care because the assistance user controls how, when, and by whom they are supported. The relationship between personal assistants and assistance users is fundamental to ensure self-determination in everyday life (Giertz, 2012). However, a well-functioning relationship between the assistance user and the PA is required. Assistants take on different roles for assistance users (Guldvik et al., 2014). Due to the interpersonal dynamics of PA, which can be characterized as a “hybrid form of work and care” (Ungerson, 1999, p. 538), some assistants consider the relational aspects as the most challenging parts of their work (Egard, 2011). PA involves inherent tensions and ambiguities: part personal, part professional; instrumental, yet at the same time emotional (Porter et al., 2020). Power is relational in the relationship between the assistant and the assistant user. Previous studies have recognized tensions about different roles and expectations, whether it be “paid friends” or “professional friendship” (Larsson, 2004; Christensen, 2012; Hultman et al., 2017, 2023).

### 1.1 Negotiating PA in the backdrop of austerity measures in Sweden

Traditionally, Sweden has had a high standard of social welfare to support people against social risk. Austerity measures in social welfare are changing the direction of social policy (Järkestig Berggren et al., 2021), for instance, when cutbacks are justified by the framing of PA as a “cost problem” (Altermark, 2017). Since 2014, policy decisions have dealt with how the costs of PA can be reduced. In the 2016 regulation letter to the Swedish Social Insurance Agency (SSIA) (Ministry of Social Affairs, 2015), the SSIA was instructed to slow down the cost development for the provision of PA.

Consequently, these austerity measures have created a debate regarding society's support for disabled people, whereby rights are being renegotiated or eroded (Ehliasson and Markström, 2020). The National Board of Health Welfare (2024) has established that several aspects indicate a worrying development that harms the quality of life and health of disabled people and their families.

Encounters between people seeking disability support and professionals are infused with routinized, invisible epistemic injustices, such as privileging professional expertise over experience-based knowledge of people with their own experiences (Carel and Kidd, 2017). Instead of focusing on its core mission, establishing a relation to the applicant, to enable a fair social needs assessment that focuses on the applicants identified needs and wishes, employees and managers in public welfare organizations often spend a considerable part of their working time on different forms of administration. Detailed control and formalism sometimes make cooperation difficult, contributing to service users with complex needs not always getting the help they need (Bringselius, 2017).

In Sweden, support and service for disabled people are provided under the Act concerning Support and Service for Persons with Certain Functional Impairments, known as the LSS Act (SFS, 1993). In LSS, it is central that disabled people are recognized as citizens and are assured equal rights as other people in society have (e.g., Grunewald, 2008). To apply for PA, the applicant must make an oral or written application and provide a detailed description of support needs in terms of the type of support needs, frequency, and duration. Needs are divided into “basic needs” and other needs, which are defined as needs connected to integrity-sensitive needs, which entail support in relation to meals, personal hygiene, dressing, undressing, and communication. Since the LSS Act came into force, additional basic needs have been added (Ministry of Social Affairs, 2022). When the granted assistance hours exceed 20 h per week, the assistance user is more likely to receive enough support to engage in leisure activities. However, if < 20 h of assistance per week are granted, those hours might not cover more than assistance to fulfill basic needs.

Over the years, government reports have repeatedly drawn attention to SSIA's difficulties in operationalizing the LSS Act. Research implies a shift from the idea of PA as a social right for citizens toward a medical model (Brennan et al., 2016) where PA resembles medical care rather than activities fulfilling policy goals such as equality and full participation in society (von Granitz, 2022). Due to the ongoing medicalization of PA, some assistance companies downplay the difference between demand-driven and supply-driven services, which implies the abolishment of user control (Ratzka, 2017).

## 2 Theoretical frameworks

### 2.1 Emotion management, emotion work, and feeling rules

Emotions are not simply an expression of individual experience. They also express collective and institutional experience (Morrison, 2007) since they are deeply embedded in and influenced by the broader social context and changes in the welfare state (Turtiainen et al., 2022).

Collins (2004) indicates how power and status affect people's ability to express emotions. Power positions and interaction create complex emotions where the actors share emotions but from very different positions. Even when the professional (the person in a superior position) understands and feels the fear of the assistance user (the person in a subordinate position), it is not the same fear that the subordinate person experiences.

Emotion management and feeling rules are focal conceptual lenses for exploring the intra- and intersubjective dynamics of people living with and being dependent upon access to PA, and people who are either making decisions about access to PA or providing PA. Instead of viewing emotions as irrational, Hochschild (2012) argues that they are subject to rules and norms, much in the same way as other behavior, which “govern both the display and the experience of emotion. Feeling rules tell us not only what emotions we should feel but also how long and how intensely we should feel them” (Lively, 2006, p. 570). The self-regulatory process of emotion management is guided by formal and informal internalized feeling rules to achieve desired emotional responses. Both emotional labor (formally internalized feeling rules) and emotion work (informal feeling rules) require a person to manage a wide range of feelings and become aware of which situations call for specific emotional responses. Those situations demand that people actively manage emotions by ensuring that their response is appropriate to the situation at hand (Lively, 2006). The emotion management perspective fosters attention to how people try to feel, not how people try to appear to feel or unconsciously feel. Emotion management is described as a behavior where “the interactive account of emotion points to alternate theoretical junctures-between consciousness of feeling and consciousness of feeling rules, between feeling rules and emotion work, between feeling rules and social structure” (Hochschild, 1979, p. 560). Influenced by Goffman (1956) Hochschild (1979) distinguishes between surface and deep acting. In surface acting, the facial expression or the body's posture feels “put on”; it is not “part of me” in contrast to deep acting, where thoughts and memories are manipulated to make feelings correspond to social norms (Lively, 2006).

### 2.2 Emotional injustice

Emotional injustice occurs due to social norms that impact the treatment of emotions (Jaggar, 1989; Ahmed, 2004; Cherry, 2019). Within Western culture, people have often been encouraged to control or suppress their emotions (Jaggar, 1989), since the inability to manage emotions has often been associated with members of subordinate groups, such as women (Cherry, 2019). For Ahmed (2004), emotions are “intentional in the sense that they are ‘about' something; they involve a direction or orientation toward an object.” Emotions always imply an act of interpretation: The “aboutness” of emotions involves a way of apprehending the world. Accordingly, when people express certain emotions, they will be perceived as having no rational (or moral) ground to have them.

In our paper, we draw upon the definition of emotional injustice coined by Pismenny et al. (2024), whereby emotional injustice is understood as an arbitrarily imposed disadvantage, i.e., features of a person or situation that are morally irrelevant or fail to justify the disadvantage or mistreatment. Emotional injustice can involve material resources, opportunities, dignity, status, free expression, and decisional capacities. Emotional injustice occurs when people in a privileged position use emotions to treat people unjustly or when the treatment of the emotions is unjust (Pismenny et al., 2024). The concept of emotional injustice has been operationalized as a taxonomy consisting of seven different categories of emotional injustices: misinterpretation, emotion discounting, extraction, emotional policing, exploitation, inequality, and weaponizing. This paper focuses on emotion discounting, an emotional analog of testimonial injustice (Fricker, 2007). One example of emotion discounting is emotion invalidating when one's responses are taken to lack credibility or worth, for example, women's anger is typically dismissed or deemed illegitimate because of the stereotype that women are “emotional” (Cherry, 2019). Another example in this category is emotion defaming, which relates to the concept of dynamic hermeneutical injustice, in which there is an intention to misrepresent (Medina, 2012). As Pismenny et al. (2024) pointed out, both misinterpretation and emotion discounting involve responses to emotions after they occur. Another category that becomes relevant for studying the intersection of disability and emotions is unjust emotional policing that underlines normative assumptions about emotion management. Emotional policing involves determining what emotions people are allowed to express, affecting their shape. One aspect of emotion policing is stereotyping, which informs our beliefs about people and can contribute to emotion misinterpretation. Stereotypes also play a role in governing the emotions of disabled people (see also Eickers, 2023) where the concept of “super crip,” contributes to the expectation of emotion work, i.e., suppressing negative emotions so those are aligned with behaviors corresponding the expectation of the “super crip,” namely, overcoming adversity and being inspirational.

### 2.3 Epistemic injustice

The concept of epistemic injustice, theorized by Fricker (2007), refers to a form of direct or indirect discrimination arising from identity prejudice of marginalized groups. When individuals or groups in society are not being listened to, nor asked to present their thoughts and experiences in matters that profoundly impact their everyday lives, they are exposed to testimonial injustice, which is one form of epistemic injustice (Fricker, 2007). Unequal power relations make disabled people vulnerable to the arbitrariness of professionals' judgments and changes in policy and legislation. It undermines the status of individuals or groups as epistemic agents (Fricker, 2007)—their capacity to act and be accepted by others as “knowers.” Fricker (2007) identified two forms of interrelated epistemic injustice. The first form, “testimonial injustice,” refers to situations in which individuals' knowledge or interpretation of events or experiences is unduly dismissed because their credibility is deflated due to prejudicial beliefs about some aspects of their identity. The second form of epistemic injustice is hermeneutical injustice, in which the actions of prejudice contrive to undermine the ability of a group of people to contribute to the collective “pool of ideas” in a society for making sense of events or an aspect of human experiences (Fricker, 2007). Hermeneutical injustice occurs when specific experiences are difficult to mediate due to a lack of a common language that makes it possible to describe a specific type of social experience that makes those experiences comprehensible to others and oneself. The possibility to describe specific social experiences entails the need for epistemic tools to perceive, describe, account for, and evaluate experience, including “language to formulate propositions, concepts to make sense of experience, procedures to approach the world, and standards to judge particular accounts of experience” (Pohlhaus, 2012, p. 718). Those in power accumulate and perpetuate power and resources for their benefit (Payne, 2002). Because language is not always seen as a means of power, its influence may go undetected by those with less power.

### 2.4 Normative life course, crip time and linked lives

The need for PA makes it more challenging to follow a normative life course. Crip time highlights the connection between following a normative life course and the ability to live according to a normative perception of time. However, living with PA destabilizes notions of normative time. For assistance users and family members, negotiating needs and wishes becomes difficult, creating linked lives between parents and disabled grown children.

The notion of a normative life course is based on a normative perception of time, chronological sequence, and particular bodies and minds (Wälivaara and Ljuslinder, 2020). In addition, a normative life course implies a linear development from childhood, adolescence, and adulthood that includes specific life events (Kafer, 2013). These life events are also structured in time to occur in a specific normative order, such as getting an education and a job, finding a partner, getting married, and having children. Crip time (Kafer, 2013) is an analytical concept that creates an understanding of time that differs from ableist time, an understanding that make us aware of the entanglement of time and the ability to follow the normative life course. Since time intersects with the life course, it shapes social norms about appropriate transition points, which contributes to creating a vulnerable life situation for disabled people who are unable to live according to normative time.

All lives are not linear yet still living in crip time challenges normative notions of straightforward time. Kafer (2013, p. 34) describes crip time as extra time, and as a departure from straight time, “whether straight time means a firm delineation between past/present/future or an expectation of linear development from dependent childhood to independent reproductive adulthood” Contrary to normative perceptions of time, crip time destabilizes normative notions of time and pace. It includes ways of being in and moving through time which are distinctly crip (Sheppard, 2020). Crip time means having both a flexible standard for punctuality and the extra time to arrive or accomplish something (Kafer, 2013, p. 26) contrary to normative time, which requires to be at the right time and use the right amount of time. Implying being “too slow, too fast, too uncontrolled, too reliant, too different, too much and also not enough” (Sheppard, 2020, p. 39). In the words of Samuels (2017, n.p.) crip time has its inherent logic:

For crip time is broken time. It requires us to break our bodies and minds to new rhythms, new patterns of thinking, feeling, and moving through the world. It forces us to take breaks even when we do not want to, even when we want to keep moving. It insists that we listen to our bodyminds so closely, so attentively, in a culture that tells us to divide the two and push the body away from us while pushing it beyond its limits. Crip time means listening to the broken languages of our bodies, translating them, honoring their words.

Living with a disability shapes the individual's subsequent life course in terms of choices, opportunities, and pathways that are either followed or expected. It also shapes the trajectories of those closely linked to the disabled person. Being dependent on others makes it more difficult to display negative emotions, such as anger, resentment, or sadness (Hultman et al., 2023).

Erickson and Ritter (2001) suggested that managing anger and frustration is a form of emotion work likely associated with increased feelings of inauthenticity. The linked lives perspective (Elder, 1998) makes ripple effects across the entire family visible. For instance, when one family member experiences stress, other family members are also affected—even if individual family members lead independent lives (Nair et al., 2022). In addition, life course trajectories that deviate from the normative life course can lead to stigmatization or even social inequalities (Ljuslinder et al., 2020).

## 3 Method

Autoethnography aims to systematically describe, analyze, and connect personal experiences to the broader social context (Ellis et al., 2011), with the researcher occupying the unique dual roles as both the object of, and the subject undertaking the investigation. Like others (e.g., Chang, 2016; Griffin and Griffin, 2019), we have tried to combine elements from different autoethnographic approaches; the “analytic” approach, to ground the findings in context (Anderson, 2006), and the emotive “evocative” approach (Ellis and Bochner, 2000), to facilitate greater understanding and evoke emotions. The continuous struggle in our everyday life, and our previous experience of writing an article about mental health care practices (Hultman and Hultman, 2023), inspired us to conduct a collaborative autoethnography (Anderson and Fourie, 2015) that enabled us to “keep our voices while creating a collective one” which offered a richer account of our experiences' (Lapadat, 2017).

Our personal experiences may differ from the experiences of other assistance users and family members. We treat our subjectivity as an approach to understanding our ways of knowing while exploring what living with PA entails. The fact that one of us holds a faculty position as a disability researcher in the global north has provided us with a “voice.” Thus, we have an epistemic privilege compared to other disabled people relying on daily assistance, whose stories remain untold due to a lack of financial and hermeneutical resources such as funding and knowledge of academic language and writing processes. Therefore, utilizing our epistemic privilege is justified because it enables us to provide an inside perspective on issues of epistemic and emotional injustices that need to be addressed.

In this study, we are bound by our mutual experiences of receiving disability support, sharing the role as supervisors for PAs, and negotiating support from professionals in charge of PA schemes. Nevertheless, as mother and daughter, our experiences differ. One of us, the daughter (Maya) is a young disabled woman — a community researcher with own experience of cerebral palsy and living with PA, and the other (Lill) is a single middled-aged woman with two children, without own experience of a mobility impairment, with a background as a social worker and disability researcher.

Critical reflexivity was applied throughout the process and was fundamental to our interpretations, which were conducted in a “back-and-forth movement between experiencing and examining a vulnerable self and observing and revealing the broader context of that experience” (Ellis, 2007, p. 14). We have explored our experiences from our differently situated knowledge (Harding, 1991). It underlines our different perspectives on handling the presence of PAs in our everyday life. Encountering each other's storying has resulted in a gradual restorying and understanding of our experiences. In this text, we utilize our positions (as people with lived experience and knowledge of theoretical concepts) as a vehicle for change by highlighting the social injustice that people needing PA may encounter. To mitigate hermeneutical injustice among ourselves, we utilize the method of talk/writing, i.e., the first author (Lill) writes while the second author (Maya) talks and is not allowed to interrupt or ask clarifying questions until the second author is finished. The initial text was written in Swedish, and we have discussed and agreed upon the theoretical concepts included in the deductive analysis we conducted together.

The analysis began with the second author identifying critical incidents, i.e., Critical Personal Narratives (CPN). For this paper, we have generated six CPNs that highlight our intertwined personal experiences. Based on these CPNs, we discussed our experiences and the relevance of our varying emotional responses to living with and being dependent on PA in everyday life. The first and second CPNs are written from Maya's perspective, and the third and fourth CPNs are written from Lill's perspective. The fifth CPN reflects Maya's perspective, and the sixth reflects Lill's. Combined, all the CPNs reflect our different but interrelated perspectives. The selected situations are used to criticize, analyze, unsettle, and defamiliarize what is often passed off as the ordinary, everyday life routines (Chapman, 2004). The narratives illustrate critical incidents involving PAs and assistance coordinators at the assistance companies involved in providing PA in everyday life. The second step was to create themes based on the chosen CPNs and analyze them deductively by utilizing concepts such as crip time, epistemic injustice, emotional injustice, emotional labor and emotion work.

We did not apply for ethical permission to conduct this study since the data consists of a text-based analysis of our personal narratives. As authors and participants, we both agreed to share our personal reflections and thoughts with each other.

## 4 Findings

Based on the CPNs, the following themes emerged: *The interconnectedness between emotion invalidation and crip time* (Section 4.1), *The expectation of emotion work* (Section 4.2), and *Managing conflicting needs in the light of emotion work and linked lives* (Section 4.3). The themes illustrate our separate and mutual voices.

### 4.1 The interconnectedness between emotion invalidation and crip time

My municipal assistance company coordinator says I must think about not using my PA at night. Because then I will not have enough hours to use the following day. She continues, by saying that: “she knows that I only use PA at night when I have to go to the hospital,” and she insinuates that I do that too often. I respond that I only go to the hospital when it is necessary, and add: “according to my neurologist, I have migraines with aura, and it could be dangerous for me to have migraines for too long.” She interrupts me and questions why migraine attacks must happen at night. I try to explain that I can't help it. What bothers me the most is that she tries to tell me what to do. She cannot possibly know how my body works. I desperately want to end the conversation, but before she ends the conversation, she says: “It will be a problem if you run out of assistance hours.” It almost makes me doubt myself – Am I making the right decision? Do I have the right to make the decision that I'm making? (Maya)

For Maya, the consequences of living with cerebral palsy fluctuate over time and can vary depending on the situation and context. During cold weather and stressful situations, her body responds with high levels of pain. She becomes more tense and sensitive to pressure. Even though she has lived with cerebral palsy all her life, her lived experience is disregarded. Thus, a nondisabled person defines what is considered a legitimate need for her. She doubts that the assistance coordinator understands varied and variable needs and how this affects the everyday lives of disabled people. It makes us think of Alison Kafer, quoted by Samuels (2017), “rather than bend disabled bodies and minds to meet the clock, crip time bends the clock to meet disabled bodies and minds,” hence, the jerky experience of living with cerebral palsy implies living in broken time—needing “extra time” for medical appointments. During specific periods, Maya's increased medical needs demand frequent hospital visits. The difference between crip time and normative time makes it difficult for Maya to translate her lived experience of variable needs neatly into a PA scheme. Contrary to a simplistic view that relies upon the proposed binaries of disability and non-disability, disabled people, like her, experience disability as fluid, which implies varied and variable needs.

Since Maya's decision regarding the provision of PA does not allow her to have assistance hours for active and practical support during the night, she risks having a shortage of assistance hours since the provision of assistance hours is based on “ideal situations” (normative time and normative needs). Thus, when there is a deficit in assistance hours, she must use assistance hours allocated for other needs or activities. Maya has to consider the practical consequences of utilizing assistance hours to which she is not entitled. In the short term, this means that she does not receive care, which can have negative health consequences in the long term. If Maya receives care, it means that there is a shortage of allocated time, which contributes to her not being able to participate in social activities. Being aware of negative consequences makes it difficult for Maya to be honest with herself. In addition, it creates a feeling of anxiety because it is impossible to make the “right choice.”

Even though the assistance coordinator has no formal power to decide how allocated time is utilized, Maya seeks her approval. To avoid emotion invalidating, it becomes important for Maya to justify her emotions by formulating arguments in a nonaggressive way. Nevertheless, anxiety connected with not being heard, or having one's emotions dismissed due to lack of credibility, makes Maya angry and fearful. At the same time, she knows that she must hide her authentic emotions since showing emotions such as anxiety and anger that are perceived to overrule normative feeling rules, connected to gender roles will only diminish her capacity as “a knower.” Emotion invalidation happens when what we do or say is not taken seriously, not taken in context, or not taken for its intended meaning.

To strengthen her epistemic agency, Maya ignores her bodily symptoms and suppresses her emotions, which creates a dissonance that makes it necessary for her to perform emotion work. If she admits her authentic feelings, it increases the amount of internal stress, reinforcing the dissonance between what she experiences and what she perceives that the assistance coordinator wants her to feel, which exemplifies emotional policing. When she adjusts her physical and emotional experiences to fit with normative expectations that are grounded in the idea of the “overcoming adversity” narrative, she learns how to distrust her feelings and ignore her own needs, which makes it easier for others to ignore her feelings (emotional discounting) as well as material needs which reinforce testimonial injustice. This policing of emotional expression can cause serious epistemic harm, both in how it influences what we define as credible testimony and in how confident we can be in the reality of our own lived experiences.

The disqualification of her lived experience and her need for hospital care that demands the presence of personal assistants exemplifies how she is wronged in her capacity as “a knower.” Acts of testimonial injustice may be described as involving disrespect and disesteem simultaneously or separately. It starkly contrasts how it feels when 'She is safe'- sharing her experience with people who validate it and express gratitude to access experience-based knowledge grounded in an inside perspective. As a minority group (Botha and Frost, 2020), there is a risk of not valuing one's perspective, which includes downgrading other people with similar experiences, as a kind of internalized ableism (Kumari Campbell, 2008).

Because of the assistance coordinator's disbelief, Maya eventually becomes silent, reluctant to continue sharing her lived experience since it becomes impossible to mediate experiences to someone who does not validate one's emotions or want to understand or consider varied or variable needs which could be understood in terms of people having different energy levels or non-normative perceptions of time. Since Maya's experiences are not considered common knowledge, the lack of legitimate concepts invalidates her narrative regarding testimonial and hermeneutical injustice (cf. Fricker, 2007). In addition, she suppresses feelings of anger and hopelessness. She cannot risk upsetting the assistance coordinator with her, since she depends on the assistance coordinator's goodwill, her being the link between Maya and her PAs. Maya perceives that she is expected to suppress anger, being able to formulate her opinions in a calm voice, without hurting other people's feelings. If she develops a poor relationship with the assistance coordinator, she risks being perceived as “difficult”, which could lead to a lack of support from the assistance coordinator. Since the assistance coordinator represents the formal employer (the assistance company) this role requires the ability to balance Maya's interests and the interests of the PAs that work with her.

My phone is ringing. It is my coordinator at the assistance company. I answer even though I'm too tired to answer. She speaks fast, and I speak slow. She says, “If you are ever mean to your personal assistant again and say you do not want to see her. She can go home, and I will send a substitute.” I try to explain that I didn't mean what I said. She briefly replies that she understands that I get upset. I notice she does not seem to understand what it is like to be upset and say something you do not mean. I say: I cannot bear to keep talking to her because she does not seem to understand me. She replies that we must continue this conversation. I listen to her and respond to the best of my ability. I feel like I want to be able to promise that I will never say something that I feel without considering the consequences it may have for others. But the question is, does she understand why I lose my temper sometimes? Because I often feel pressured, I swallow and swallow, and to avoid assistants questioning my decisions, I let them choose when things should be done and sometimes how things should be carried out. I do this because I depend on the assistants all the time. I swallow and swallow, until I can't take it anymore. (Maya)

In the conversation with Maya, the assistant coordinator takes on the dual role of employer and “knower.” The coordinator seems to ignore the essential difference between being a PA and someone needing a PA. For the assistants, it is a workplace. When they end their shift, they have their place to go, where they can relax, choose to be alone or socialize with friends, without someone else being present. For Maya, it is her private space and sanctuary. It is where she should be able to be “backstage,” not having to perform a role or have the ableist gaze bestowed upon her.

Instead of acknowledging Maya's emotions regarding the difficulty of having a PA present around the clock, the assistance coordinator wants to find a quick solution and possibly a scapegoat. When there is a disagreement between Maya and one of her PAs, Maya often feels that the assistance coordinator sides with the PAs, instead of being neutral and listening to both sides. Maya experience that the assistance coordinator blames her for being “difficult and demanding”, that she should be able to do emotion work and obey feeling rules, since displaying strong emotions such as anger is considered an “inappropriate response” contrary to the idea of women being sweet and considerate of other people's feelings. Being dependent on maintaining good relations with PAs makes it difficult for Maya to display authentic feelings. Therefore, she tries to suppress the anger and disappointment felt toward her PAs. By engaging in surface acting, Maya tries to adapt her emotions and behavior to other people's expectations.

### 4.2 The expectation of emotion work

For Lill, contact with different assistance providers evokes conflicting emotions. On the one hand, it feels like an obligation to secure her daughter's right to obtain high-quality PA and to ensure that the assistance company fulfills its duties. On the other hand, she is tired of being involved in all aspects of her daughter's life. It feels like some professionals think she is unwilling or unable to allow her daughter to become independent since there is a general misconception that parents of disabled children are being overprotective (Holmbeck et al., 2002).

Sometimes, I am afraid of being perceived as unreasonable or “a know-it-all” and that my involvement might backfire and reduce my daughter's chance of gaining access to PA according to the intention in the LSS legislation. Depending on which professional I meet, I could be cast as the overprotective, heroic, or selfish mother. At the same time, speaking for oneself and utilizing the same language as professionals makes it easier for us to gain access to support. I suspect that if I have a nervous breakdown during an assessment meeting, I'd probably get more sympathy and less power. I often get frustrated that we must fight for our rights. The struggle never ends. It is so exhausting, frightening, and overwhelming that professionals have so much power over our everyday lives. It is so unfair. Over the years, I have become a warrior. I feel that being in touch with my anger has helped me continue fighting for our rights. At the same time, awareness of the discrepancy between policy and practice has created enormous feelings of hopelessness. (Lill)

Being squeezed between different expectations from others and her own needs, working full time, having “me-time” to recuperate, tending to household chores, and being a “good mother” to siblings. Lill often feels that she is expected to do emotion work. Cast in the “good mother” role, she experiences herself being restrained by feeling rules that expect her to provide accurate and nuanced descriptions of her daughter's needs in a neutral manner or possibly display feelings of acceptance or sadness. When she fails to display “the correct emotions,” by neither complying with feeling rules nor gender roles, i.e., displaying anger instead of maintaining her composure, she has experienced that some of Maya's assistance coordinators have expressed their disappointment in her. They expected her to do better, i.e., to be “professional” and act as a “role model” for personal assistants. This creates internal stress, as it is difficult for her to perform surface acting which is reinforced by the fact that she is aware that her ability to control her emotions can affect if professionals perceive her as knowledgeable. When she can be both determined and friendly, she stands a better chance of advocating for her support needs.

Contrary to Lill's own beliefs, some health care professionals attribute her stress to Maya's disability, according to narratives framing disability as a personal tragedy. Denied epistemic agency can be understood as a combination of epistemic and emotional injustice exposure. Even when different professionals say that they understand that her anger and frustration are rooted in an overwhelming life situation, it does not change the fact that she feels obliged to act according to gendered feeling rules, such as trusting professional judgement and being greatful for the support received. Since Lill knows that she is feeling something in opposition to what she is “allowed” to feel, she tries to regulate her expression by adapting her presentation of emotionally charged information so that the intended audience, i.e., professionals, will feel more comfortable with what she is saying. She cannot risk jeopardizing access to support and the quality of the support provided. In this situation, Lill perceives that the existence of “socially unacceptable emotions” in her testimony undermines the validity of all components of the testimony, including the reason or fact-based aspects, even when they are entirely relevant and appropriate to the context of the testimony.

Being dependent on others to get to work creates stress. Lill can recall many times when PAs have not arrived on time, and she has been unable to leave home until they arrive. She wishes it were not so obvious how she feels in such situations, as it only makes things worse both in the short term and in the long run. Making the PAs feel uncomfortable can make future interactions difficult, especially when there is no time to talk things over and things are left unsaid.

When I am stressed out, I cannot display a poker face and express myself in a polite manner. How practical it would be if I could quickly switch to a more neutral state of mind, instead of being upset. The chronic stress of constantly being forced to be in a stand-by mode sometimes makes me react this strongly. It probably seems unreasonable to a person unaware of the “big picture.” I do not want to feel like this. I want to relax, feel safe, secure, and content with my life. I wish I did not have to be around unfamiliar people, unknown bodies, and voices. It feels like our house has revolving doors, and sometimes I get the urge to hide in my bedroom, which I sometimes do. However, then I feel like I am being unfair, and ungrateful, because when PA works as it should, it is a relief for all of us. It allows us to live our separate lives according to our own choices – to do all the things most people take for granted; to work, study, be spontaneous, and meet friends. (Lill)

Even though Lill has empathetic colleagues at work, it is difficult to explain that gaining access to PA is not the same as having well-functioning assistance in everyday life. In periods of their life when there has been a high rate of staff turnover, it has had an immediate impact on her involvement in care work, which affected her being on time at her regular job. Hence, the broken time (Samuels, 2017) also becomes her time. Lill fears the consequences of departing from the normative life course, even though she is tired of being worn out and constantly worrying about Maya. Discussions with colleagues sometimes feel superficial. On the one hand, she wants to be authentic and able to talk about her family life, including living in a vulnerable situation. On the other hand, she is tired of focusing on challenges and hardships and explaining her situation to people unfamiliar with her circumstances. These mixed emotions make her feel obligated to obey feeling rules, such as having a positive attitude, being focused on not taking up too much space, and being considerate toward other people's emotions and well-being since she does not want to make anyone else feel uncomfortable or stand the risk of being perceived as an object of pity. Sometimes she becomes envious of colleagues with grown-up children, since this enables them to prioritize their own needs. Some days her major fear is to leave her professional job and identity, becoming isolated at home, or being reduced to being the primary caregiver. It becomes an impossible equation to balance her needs with different family members' needs, and still, that is what many parents with disabled children must cope with.

### 4.3 Managing our conflicting needs in the light of emotion work and linked lives

For the assistance user and other family members, access to PAs is a prerequisite for living independent lives. The absence of PAs creates a stressful situation for the entire family, and it can contribute to strained relations between different family members. The occurrence of linked lives can create a situation where we experience mutual lock-in effects that create feelings of guilt and frustration. Being forced into the roles of assistant and assistant user makes it difficult to appreciate each other's company. The relationship between PAs and assistance users is asymmetrical. It is a professional relationship where PAs and assistant users must maintain a professional yet friendly relationship. This role expectation can become complicated when the assistant is a close relative since the relationship is more complex, and there can be a higher expectancy of reciprocity.

Periodically, I have had assistance where I felt like a person of my own age, free and independent. When it does not work, I feel locked in. I become stuck in a way that reduces my identity to being an assistant user. I only get one type of relationship: I become the person who receives support, and the other person gets reduced to someone who provides support. It feels like I've taken up too much space. (Maya)

For Maya, it creates an experience of being off time. Being dependent on support from her mother creates a situation that is more like what she experienced as a child. It becomes emotionally challenging to have those dual roles of being mother/daughter and PA/assistant user, which highlights our conflicting needs. It is accentuated by Lill having to cover up for external assistants when they are absent. It makes it difficult for Maya to plan her time and makes her feel guilty for Lill having to put other tasks aside, even when she does not have the time. It makes both of them miss many parts of what is perceived as ordinary, following a normative life course, such as dating, going to the pub, or haning out with friends.

Being forced to be with each other around the clock dulls even the fun things. Then it is easy to forget that we enjoy each other's company. Sometimes it feels like the assistance company takes advantage of me and ignores our needs and wishes. It creates a lot of ambivalent feelings, especially when I feel like I should support Maya, but I really can't. Then I feel bad, but I'm afraid of what will happen the day that I am too exhausted. It's unfair because neither Maya nor I can choose how we want to live. There is such a big difference when the assistance works as it should, it is like night and day. (Lill)

As a parent, Lill often thinks this is the last time she will “work” as her daughter's PA. Lack of external PAs makes it difficult to set “healthy boundaries”. Being able to choose each other's company rather than being forced to interact would strengthen the ability to create a more symmetrical relationship. When we cannot “choose each other”, the levels of mutual frustration increase since we cannot leave each other and go home because we are already at home. We are still stuck in the same physical and emotional context.

## 5 Discussion and conclusion

In this article, the aim was to explore the emotion work around the navigation of emotional and epistemic injustice faced by disabled people and their family members, which is exemplified by utilizing our own experiences of living with PA in everyday life. Unequal distribution of social power is salient both in the process of applying for PA and the implementation of PA in everyday life. As Tremain (2017) pointed out, certain forms of unequal social power produce disciplinary norms about proper social behavior that shape public perceptions and authoritative epistemologies. A person's social position dictates how and to what extent they can express their emotions. If an individual fails to consider these social rules, they risk losing their credibility as an epistemic agent, which involves defining the reality of their own experiences. When assistance coordinators fail to acknowledge the lived experience of disability and have normative ideas of what the relationship between PAs and assistance users should entail, it leaves little room for developing an authentic relationship between the assistance user and individual PAs. Being dependent on maintaining a good relationship with PAs, social workers, or health care staff (see, for example, Hultman and Hultman, 2023) makes living with PA emotionally challenging.

Contrary to a nondisabled person the disabled person must navigate challenges related to crip time (Kafer, 2013). For example, there is a need for more time to accomplish tasks and duties that are usually easier and faster for non-disabled people. Lack of understanding the consequence of living in crip time, assistance users and their family members experience a need to perform emotion work both about external PAs and about the assistance coordinator. Previous experiences of non-disabled people's lack of understanding the consequences of living with crip time makes Maya inclined to justify her fluctuating assistance needs. To maintain a positive relationship and to protect herself from criticism and discomfort she tries to talk about it in a detached, unemotional way according to emotionally detached (normative understandings) of professional relationships.

The complexity of our everyday lives can make it difficult for professionals to consider the impact of linked lives (Elder, 1998) and the potential adverse outcomes. Due to the emotion work needed to assume different roles vis-á-vis each other, i.e., we are bound together by affection (as mother and daughter) and by necessity (as an assistance user and PA). Ambiguous roles can create conflicting needs and harm long-term health and wellbeing, as societal expectations and a shortage of external PAs pressure both assistance users and family members to assume the roles of PA and assistance user. Around the clock, different types of support are provided (attending assessment meetings regarding access to PA, health care meetings, collaborating with the assistance coordinator, working as a PA, providing emotional support), equal extended care. Care that goes beyond what one would expect as a mother due to professionals' expectations of mothers' moral commitment to take on a caring persona (Rogers, 2012). As a moral expectation, this requires linked lives (Elder, 1998), incompatible with the normative idea of independence and a need for separate lives.

Our sense of who we are and what we can achieve as epistemic agents is continually (re)shaped by how we feel (Davidson and Milligan, 2004). Having external PAs in one's home environment creates a sense of being unable to escape either emotionally or physically, which makes it important to develop authentic relations with external PAs and coordinators since the lack of authentic relations underscores the felt pressure of having to perform emotion work.

When emotion work fails because a tipping point has been reached, our positions as epistemic agents are questioned. It exemplifies emotional invalidation, the emotional counterpart to testimonial injustice. Being dismissed as “a knower” (Fricker, 2007) can create feelings of self-doubt, in which the assistance user values the opinions of non-disabled professionals more than lived experience. Not being validated and heard makes disabled people and allies (such as family members) more vulnerable to normative opinions about what is considered legitimate needs or an emotionally appropriate behavior. Epistemic injustice is often enacted in micro-meetings, such as relations between assistance users and PAs. However, these harmful actions often derive from epistemic practices which can be found on a structural level (Dunne, 2020).

The felt need to perform “balanced emotions” (surface acting) (Hochschild, 1979) could be seen as an attempt to convince the assistance coordinator and external PAs of the legitimacy of expressed needs and wishes. All three themes exemplify the presupposed binary between rationality and emotionality, where both Maya and Lill are exposed to an emotional double bind where they either must redirect energy to the regulation of intense emotions to have a better chance of being heard, and risk, emotional dissonance and depersonalization, or express their authentic emotions while speaking on a personal experience of oppression and risk being dismissed as overreacting.

This emotion-regulation double bind is reflected in Bailey's (2018) work on silencing spirals. As Bailey (2018) notes, these silencing spirals are a “closed hermeneutical system” in which the speaker suffers a double epistemic injustice—neither the testimony nor the authentic emotions are validated. This occurrence of both epistemic and emotional injustice builds with each layer of demands from people in “dominantly situated positions,” such as assistance coordinators and external PAs. When assistance coordinators or external PAs require certain emotions to be removed from the experience of assistance users or family members, for it to be seen as credible, they fail to recognize the value of epistemically relevant information about a situation. Silencing people with lived experience creates a situation where people with insight into an injustice are those most likely to become emotional while talking about it, and therefore more likely to have their relevant first-hand knowledge dismissed (Whalley, 2022). With this silencing cycle, those systems of oppression and dismissal continue, and the instances of epistemic injustice remain intact. By defining and analyzing this emotion-specific form of epistemic injustice, we can begin to value emotions as a powerful resource for real social and political change.

## Data Availability

The datasets presented in this article are not readily available because the study is based on personal narratives that are already included in the manuscript.
